# Physical and Behavioral Measures that Predict Cats’ Socialization in an Animal Shelter Environment during a Three Day Period

**DOI:** 10.3390/ani3041215

**Published:** 2013-12-18

**Authors:** Margaret Slater, Laurie Garrison, Katherine Miller, Emily Weiss, Natasha Drain, Kathleen Makolinski

**Affiliations:** 1 Shelter Research and Development, Community Outreach, American Society for the Prevention of Cruelty to Animals (ASPCA^®^), 50 Stone Ridge Drive, Florence, MA 01062, USA; 2 Shelter Research and Development, Community Outreach, American Society for the Prevention of Cruelty to Animals (ASPCA^®^), P.O. Box 408, Little Silver, NJ 07739, USA; E-Mail: laurie.garrison@aspca.org; 3 Shelter Research and Development, Community Outreach, American Society for the Prevention of Cruelty to Animals (ASPCA^®^), 520 Eighth Avenue, 7th Floor, New York, NY 10018, USA; E-Mail: katherine.miller@aspca.org; 4 Shelter Research and Development, Community Outreach, American Society for the Prevention of Cruelty to Animals (ASPCA^®^), 3201 SW Winding Way, Palm City, FL 34990, USA; E-Mail: emily.weiss@aspca.org; 5 Shelter Research and Development, Community Outreach, American Society for the Prevention of Cruelty to Animals (ASPCA^®^), P.O. Box 4323, Arlington, VA 22204, USA; E-Mail: natasha.drain@aspca.org; 6 Veterinary Outreach, Community Outreach, American Society for the Prevention of Cruelty to Animals (ASPCA^®^), P.O. Box 1144, Orchard Park, NY 14127, USA; E-Mail: kathleen.makolinski@aspca.org

**Keywords:** feral cat, socialization, animal shelter, behavior, cat rescue, stray cat, logistic regression, cat

## Abstract

**Simple Summary:**

Information from surveys completed by the cats’ caregivers provided a score for the level of socialization of cats. We examined the effectiveness of structured assessments and measures in their ability to distinguish More and Less Socialized cats in a shelter-like setting over a three day period. Statistical models were developed that best predicted More and Less Socialized cats. Measures from these models were used to calculate a point system where more points indicated more socialization. In combination with key socialized behaviors, these points were able to fairly accurately distinguish More Socialized from Less Socialized cats.

**Abstract:**

Animal welfare organizations typically take in cats with unknown levels of socialization towards humans, ranging from unsocialized cats well-socialized but lost pets. Agencies typically determine the socialization status and disposition options of cats within three days, when even a well-socialized pet may be too frightened of the unfamiliar surroundings to display its typical behavior. This is the third part of a three-phase project to develop and evaluate a reliable and valid tool to predict cats’ socialization levels. We recruited cats from the full spectrum of socialization and, using information from the cats’ caregivers regarding typical behavior toward familiar and unfamiliar people, assigned each cat to a Socialization Category. This information was compared to the cats’ behavior during three days of structured assessments conducted in a shelter-like setting. The results of logistic regression modeling generated two models using assessments from the mornings of the second and third day, focusing on predicting shyer or more aloof but socialized cats. Using the coefficients from each of these models, two sets of points were calculated which were useful in differentiating More and Less Socialized cats. In combination with key socialized behaviors, these points were able to fairly accurately identify More and Less Socialized cats.

## 1. Introduction

In the United States, millions of cats enter animal shelters each year [1,2]. The handling, housing, and disposition (*i.e.*, whether the cat will be considered for adoption, returned to the original location, or euthanized) of each cat depends upon the cat’s health and how well socialized the cat appears to be with people. Shelters have limited resources to devote to any single cat and being assigned to an appropriate disposition track can increase the overall likelihood for live outcomes for cats entering the shelter [3,4]. Thus, shelter staff need to determine as soon as possible, usually within a one to three day holding period, whether a cat is sufficiently socialized with humans to be considered for adoption [5].

Among the cats entering shelters, many are free-roaming or stray cats brought to the shelter by people who are not their caregivers. Therefore, relatively little is known about the cat’s history and behavior [6,7,8]. Consequently, for any free-roaming, unowned cat entering a shelter, staff must do their best to differentiate between cats that are truly unsocialized with people and those that are moderately- to well-socialized but frightened. However, there are no validated methods to accomplish this. 

Cats’ socialization with humans runs along a poorly studied spectrum from truly feral to well-socialized. Cats can also change where they appear to be along that spectrum with time, changes in environment and/or behavioral rehabilitation [5,9]. Truly feral cats were not socialized with people as kittens and will remain wary of humans throughout their lives [9]. However, even the most well-socialized, long-term house cat can display fearful behavior in novel situations and with unknown people, making it difficult to determine how well-socialized any cat truly is while in a shelter setting. Thus, in some shelters, any cats acting extremely fearfully may be deemed “feral” and possibly euthanized without being held for a period of time [5]. There are also instances where shelter staff opt to euthanize a certain cat, in spite of “mandatory” holding periods, due to the cat’s apparent lack of socialization. This also means that some frightened but well-socialized pets may be euthanized before their owners can reclaim them. In addition, many moderately–socialized, shy cats could be at a high risk for euthanasia but could have benefited from an adjustment period or behavior modification program. Conversely, in other shelters, a truly feral cat may be held longer than the standard holding period—increasing the cat’s distress—in the hopes that it will “settle down” and show itself to be a socialized cat. Holding unsocialized cats for longer than necessary has substantial welfare implications for these cats and is not recommended.

This study is the third part of a three-phase project to determine if a reliable and valid assessment tool for shelter staff can be developed to differentiate cats by socialization status within the typical shelter three day holding period. Since cat socialization is a continuous spectrum, the tool should also provide some insight into where on this spectrum of socialization this cat is likely to fall at that particular point in time. In the first phase of developing this tool, the reliability and validity of the Cat Behavior and Background survey [10] was evaluated and an overall ordinal cat Socialization Score and dichotomized Socialization Category (More Socialized *vs.* Less Socialized with humans) developed. In the second phase, a variety of structured assessments and measures were evaluated for their relationships with Socialization Category (More *vs.* Less Socialized), along with how the data varied across assessments and over time within a three day period [11]. The current study serves to: (1) determine which of those measures were significant predictors of cats’ owner/caregiver-reported Socialization Category (More *vs.* Less Socialized) in logistic regression models; (2) develop a point system based on the coefficients of these models for use in animal shelters to indicate level of socialization; and (3) describe the relationships among the affiliative and attention seeking behaviors (Behavior Checklist [11]), the point system and the Socialization Score reported by the caregiver. 

## 2. Methods

### 2.1. Subjects

This study was approved by the ASPCA^®^ Institutional Review Board and conducted from January through April 2012 at People for Animals Spay/Neuter Clinic and Rescue (PFA) in Hillside, New Jersey, United States. The owners and caregivers (hereafter referred to collectively as “caregivers”) of the cats were recruited through professional networking, online advertisements, flyers, and by phone. Cats eligible for participation were not visibly pregnant, nursing, or in obvious heat, were between 6 months and 10 years old by best estimate, and in good health. Ten years was selected as a cut off to avoid including elderly cats with subclinical disease which might influence their behavior. Cats displaying signs of illness that could compromise health or behavior at any time during the study were examined by a veterinarian and excluded. 

We encouraged the participation of cats anywhere along the spectrum of socialization towards humans, from truly untouchable, unsocialized ferals to well-socialized house pets. Participating owned or fostered cats must have been living in the home for at least 1 month; unowned, free-roaming cats had to have been seen regularly by the caregiver for at least 1 month. To limit disease transmission, owned cats and unowned cats (in foster care or outdoor homeless cats) were recruited and studied in alternating study replicates of up to 16 cats for 25 weeks. At the conclusion of the study period, all unaltered cats were spayed or neutered at no charge or for a nominal fee. 

There were 297 eligible cats from 161 caregivers. The median number of cats in the study from each caregiver/owner was 1 with a range of 1 to 7. There were 159 male cats (54%) and 138 female cats (46%). There were more owned/fostered cats (n = 161) than unowned cats (n = 136). These are the same cats as in the related paper; for a full description, please see [11].

### 2.2. Procedure and Study Conditions

Day 1 of the three day study began with admitting the cats by 11 AM. The caregivers completed an informed consent form, the clinic’s standard spay/neuter surgery intake form, and the Cat Behavior and Background Survey which described the cat’s usual behavior and duration of ownership/care [10]. Each cat was given a visual health examination, a feline viral rhinotracheitis, calicivirus and panleukopenia virus (FVRCP) vaccine, and a flea treatment. The cats were transferred in the same order that they had arrived, from their traps or carriers to individual cages in the study room which was set up to simulate a typical shelter environment. The room contained 16 stainless steel cages, arranged so the cats were unable to see each other. Each cage contained food and water bowls mounted to the left front of the cage, a litter box at the right front of the cage, a plush bed at the back under a raised Kuranda™ bed, and a wash cloth to cover the remaining cage floor. The structured assessments were performed on Days 1 through 3. On Day 4, unaltered cats were sterilized, given a rabies vaccine and returned to their caregivers later that day. See [11] for more details.

### 2.3. Cat Socialization Score and Socialization Category from Caregiver Survey

At intake, caregivers turned in their completed Cat Behavior and Background Surveys [10]. Based on the answers to the survey, a Socialization Score and Socialization Category were assigned to the cat. Each cat’s Socialization Score was calculated as the median score of the 11 reliable and valid Cat Behavior and Background Survey questions that were rated on a 0 to 10 scale. Responses of “I don’t know” and “it’s not safe to try” were treated as missing. Socialization Scores ranged from 0, which could be considered a completely unsocialized or feral cat, to 10, a very well-socialized cat. Because the goal of the project was to separate the Less Socialized from the More Socialized cats, Socialization Scores were dichotomized for subsequent analyses into a Socialization Category. The cut-point was based on the scores’ natural distribution. The dichotomous Socialization Categories created were “Less Socialized” cats (0–3) which were those at the very low end of the socialization scale and “More Socialized” cats (4–10), which were those in the middle to high end of the socialization scale.

### 2.4. Structured Assessments of Cats’ Behavior during the 3-Day Study Period

The structured behavior assessments were specified interaction with each cat that were designed to elicit responses from the cats. The assessments were conducted over three days during five time periods (PM-Day 1, AM-Day 2, PM-Day 2, AM-Day 3, PM-Day 3). During each time period, five assessments were conducted: Greet (where the observer stood in front of the cage and talked softly to the cat for 15 seconds), Crack Cage Door (where the observer says “hi, kitty” and stands with her hand on the cage for 30 seconds, then cracks the door and closes it), Novel Object (where a pair of sunglasses, clean litter box scoop or plastic cup was hung on the cage front for 30 seconds), Interactive Toy (where a string on a rod was jiggled in the front of the cage for 30 seconds) and Touch with Rod (where the observer presented a rubber-tipped rod to the cat to smell, then stroked the cat for 10 seconds, presented the rod again then pushed down on the cat’s shoulders for 10 seconds). A sixth assessment, Eating in Presence of Observer, was only performed in the PM assessments. A total of 46 behavioral, physical and environmental measures were recorded. Measures included vocalization, alertness of the cat, body, head and tail position, location of the cat in the cage, eye contact, blinking, affiliative and defensive behaviors, attention to the person, toy or rod, presence of urine and feces, consumption of food and water and messiness of the cage [11]. All assessments were conducted in person by one observer, blind to the Socialization Score but aware of which groups were owned pets and unowned cats. All assessments were video recorded for future reference if necessary.

On Day 1, after the cats had been transferred to the study room and left undisturbed inside their cages for at least one hour, the PM-Day 1 structured assessments were performed. For Day 2 and 3, the AM assessments were performed in the morning, followed by spot cleaning by the observer and adding fresh water to the bowls. The cats were left undisturbed for one hour, after which the PM structured assessments were performed. A full description of the structured assessments and measures as well as the procedures can be found in [11].

### 2.5. Descriptive Statistics and Data Analyses

Frequencies and percentages were used to summarize all categorical variables. Some variables could not be included in the modeling process due to zero values in one or more cells. A separate Behavior Checklist of affiliative and attention seeking behaviors categorized as Strong or Weak behaviors was developed [11]. Strong behaviors (one of these at any time resulted in the cat being classified as More Socialized) included rub, knead, touch, play, at the front of the cage at any time, chirp and tail up in the air. Weak behaviors (four or more at any time in any combination resulted in the cat being classified as More Socialized) included sniff, roll, reach, approach front of cage, yawn, groom/shake and standing or still moving at the end of the assessment. Many of these Checklist behaviors included variables with zero cells. Spearman rank sum correlation coefficients were used to examine the linear relationships between Socialization Score and total Strong behaviors, total Weak behaviors (both from the Behavior Checklist), and the Points created from the models (see Section 2.6.1). Ninety five percent confidence intervals for the correlations and some point estimates were also calculated. 

Each time period was examined for its usefulness in predicting socialization. We performed univariable Fisher Exact tests for the association of categorical independent variables and the dichotomized Socialization Category to identify measures that differed between “Less Socialized” and “More Socialized” cats. Standard statistical software was used for all analyses (StataSE 12 (64 bit), StataCorp LP, College Station, TX, USA).

### 2.6. Statistical Modeling

Our primary objective for these analyses was to predict the cat’s Socialization Category as accurately as possible with as few measures, assessments and time periods as possible. To facilitate this, the process for logistic regression modeling was focused on eliminating the “easy-to-identify cats”, leaving a subset of “more-difficult to identify” cats in each assessment. The Behavior Checklist was used to identify cats classified as More Socialized (one or more Strong behavior or four or more Weak behaviors at any time during the assessments) during PM-Day 1 so that these “easy-to-identify” cats were not included in the subsequent time period models (55 cats were removed). No additional modeling of PM-Day 1 was performed (the initial logistic regression model for PM-Day 1 did not perform as well as the subsequent time periods, data not shown). Cats with more than 31 points (see Section 2.6.1) on AM-Day 2 included only More Socialized cats according to the Socialization Category and the Behavior Checklist. Therefore, cats with points greater than 31 on the AM-Day 2 model were excluded from subsequent models (PM-Day 2 and following) with the goal of better determining the “more difficult-to-identify” socialized cats (the more shy or less interactive cats), a critical element for this study (52 additional cats removed). Additional removal of cats from the modeling was not performed to avoid making the sample size too small.

A separate multivariable binary logistic regression analysis for each of the remaining time periods (AM-Day 2, PM-Day 2, AM-Day 3 and PM-Day 3) was then developed using the identified subset of cats. For each assessment in each time period, variables (measures) with *p* < 0.25 on Fisher exact test were offered to the logistic regression models. We used forward stepwise analysis with a likelihood ratio test to retain the variable at *p* ≤ 0.2 for each assessment separately. Measures which were significant at ≤0.2 for each assessment were then combined into a single time period model using a forward stepwise approach and *p* < 0.05, resulting in one model per time period. Afterwards, all measures that were dropped from the models were re-entered one by one into the model and again assessed for significance. All models were tested for all possible two-way interactions. Odds ratios and their 95% confidence intervals were calculated for each retained measure in each model. 

Once final models for each time period were determined, responses to the individual measures were collapsed to create a simpler model that still did well at predicting Socialization Category. For example, the “Affiliative Behavior” variable was dichotomized to 1 = any affiliative behavior and 0 = no affiliative behavior. Categories were only collapsed as far as needed to eliminate zero cells or similar odds ratios, so the number of categories could differ in different final models. The Hosmer-Lemeshow goodness-of-fit test was examined for model fit. Sensitivity, specificity, and the Area Under the Receiver Operating Curve (AUC) were calculated assuming that the dichotomized Socialization Category was the reference standard. 

Finally, the accuracy of predicting Socialization Category for each cat was examined in detail to see if some assessments or time periods could be dropped while still maximizing the ability of the models and Behavior Checklist to correctly identify measures that included More Socialized cats (Socialization Scores of more than three). Maximizing the ability to predict More Socialized cats was selected because it appeared that More Socialized cats were identified by their showing more behaviors than Less Socialized cats. 

#### 2.6.1. Development of the Point System from the Logistic Regression Models

Coefficients from each of the final models were converted to points. This was done by rounding the coefficient to the nearest 0.5, determining a multiplier to reach a total of 50 points for all measures in the model and then, if necessary, adjusting one or two measures’ points up or down to sum to 50 for the full model. For example, a coefficient of 0.69 would be rounded to 1 then multiplied by 4. If needed, it might be adjusted to 3 if the multiplier made the total greater than 50 so that the sum of all measures’ points was 50. Table 1 includes the points used for both models. Points for each cat were graphed against Socialization Score to visualize the relationship and later provide guidance for interpreting the points. 

### 2.7. Validation of Model Data

Data were obtained from 250 cats from April through October, 2010 at the Humane Alliance Spay/Neuter Clinic (HA) in Asheville, North Carolina, United States, using the same recruitment and inclusion criteria as previously described [11]. There were four differences in the assessments for the HA data set compared to the PFA data set: (1) the Novel Object was only done during the PM time periods and the Interactive Toy only during the AM time periods; (2) each assessment was done with all cats and then the next assessment was done with all cats and so on (so all cats had the Greet assessment done first, whereas at PFA each cat had all six assessments done before moving on to the next cat). This sequence was changed at PFA to provide a longer and more consistent observer interaction to try to elicit as much response in the shy cats as possible; (3) there was a $10 incentive at a pet supply store added the third month for Less Socialized cats; (4) all specific affiliative behaviors during the assessments were individually entered into the data set which was not done at PFA. Several additional measures were used at HA but all measures in the current study were included in this validation data set. Final models from the current study were run using these HA data and the sensitivity, specificity and AUC were calculated and compared with the current study. Points were also calculated from the HA data set and examined for accuracy of predicting Socialization Category.

## 3. Results

### 3.1. Selection of Minimum Models Needed

Models using the five assessments in the five time periods were created and all cats assigned points as described. The Behavior Checklist also used data from all five time periods to identify More Socialized cats. We found that only the two AM models were needed to obtain good predictions. Based on visual analysis of graphs to balance false negative (socialized cats classified as Less Socialized) and false positive (unsocialized cats classified as More Socialized) results, the cut-off of ≥20 for the AM-Day 2 model and ≥12 for the AM-Day 3 models were determined. With these cut-offs for the two AM models and the results from the Behavior Checklist, four cats that were More Socialized were misclassified as Less Socialized. In an effort to further reduce the time and effort required to assess these cats in the shelter setting, data from the Novel Object assessment and from PM-Day 3 were dropped and the models’ predictive ability and Behavior Checklist re-examined. 

**Table 1:** Final logistic regression models without Novel Object for AM-Day 2 (excluding socialized cats based on the Behavior Checklist) and AM-Day 3 (excluding socialized cats based on the Behavior Checklist as well as cats with AM-Day 2 points greater than 31).

	Number (%) Unsocialized	Number (%) Socialized	Odds Ratio	95% CI for OR	Coefficient	Points
AM-Day 2 Model (n = 242)						
Any affiliative during Greet	3 (4)	57 (35)	9.3	2.5–35	2.2	9
Ate during the night	73 (87)	106 (65)	0.3	0.1–0.6	−1.4	7
Licked lips/nose Crack Cage Door	5 (6)	49 (30)	4.3	1.4–13	1.5	7
Head facing toward or can’t tell at end during Toy	78 (93)	159 (98)	10.1	0.9–119	2.3	11
≥50% attention to toy	46 (55)	139 (86)	3.1	1.5–6.6	1.1	5
Sniffed the rod at first presentation	49 (58)	137 (85)	2.4	1.1–5.0	0.9	4
Any affiliative behavior during Push with Rod	4 (5)	46 (28)	3.7	1.0–14	1.3	7
AM-Day 3 Model (n = 190)						
Eye contact throughout Greet ≥50% of the time	38 (45)	18 (17)	0.3	0.1–0.6	−1.1	7
Fully alert during Crack Cage Door	80 (96)	92 (86)	0.13	0.03–0.5	−2.1	9
Withdraw from person at any time during Crack Cage	2 (2)	12 (11)	8.5	1.3–54	2.1	9
Any affiliative behavior during Toy	2 (2)	25 (23)	7.2	1.5–36	2.0	9
Head position in front or middle of cage at end of Rod	26 (31)	67 (63)	2.8	1.3–5.5	1.0	5
Sniff rod on first presentation	49 (58)	84 (83)	2.7	1.1–6.3	1.0	5
Any affiliative behavior during Stroke with Rod	7 (8)	39 (37)	3.7	1.4–10	1.3	7

AM-Day 2: n = 245; sensitivity 89.44%; specificity 58.2%; AUC 0.8533; Hosmer-Lemeshow goodness of fit *p* = 0.87; AM-Day 3: n = 190; sensitivity 75.70%; specificity 80.72%; AUC 0.8477; Hosmer-Lemeshow goodness of fit *p* = 0.53.

For the final AM-Day 2 model, variables offered to the model at *p* ≤ 0.2 for each assessment were: Greet: (any) Affiliative Behavior, Lick Lips/Nose (at any time), Stretch (at any time), Eye Contact (throughout), Blinking (throughout), Approach (at any time), Ate (during the night); Crack Cage Door: Head Location (at end), Lick Lips/Nose (at any time), Tail Position (at end), Ear Position (at end), Location (at end); Interactive toy: (any) Affiliative Behavior, Head Facing (at end), Lick Lips/Nose (at any time), Attention to Toy (throughout), Location (at end); Touch with Rod: (any) Affiliative Behavior When Pushed With Rod, (any) Aggressive Behavior When Pushed With Rod, Head Position (at end), Attention to Rod (throughout), Sniff Rod on First Presentation. Seven of these measures and 242 cats remained in the model once the PM-Day 1 More Socialized cats were removed (see Table 1).

For the final AM-Day 3 model, variables offered to the model at *p* ≤ 0.2 were: Greet: Eye Contact (throughout), Drank (water); Crack Cage Door: Lick Lips/nose (at any time), Meow/purr (at any time), Alertness (at end), Tail Position (at end), Maximum Body Movement, Maximum of Head Movement, Blinking (throughout), Approach (at any time), Withdraw (at any time); Interactive Toy: (any) Affiliative Behavior, Attention to Person (throughout), Location (at end); Touch with Rod: Head Location (at end), Attention to Person (throughout), Withdraw (at any time), Blinking (throughout), Sniff Rod on First Presentation and Sniff Rod on Second Presentation. Seven of these measures and 190 cats were included in the final model once the PM-Day 1 More Socialized cats and the cats with >31 points on the AM-Day 2 model were removed (see Table 1). No interactions were significant in either model.

With these final models determining the point count for each cat, only 3 (1.4% 95% CI of 0.3–4.1%) More Socialized cats were misclassified by all three indicators (AM-Day 2 points, AM-Day 3 points and Behavior Checklist) as Less Socialized. There were 45 cats categorized as Less Socialized by the Socialization Score and the Behavior Checklist that were classified as More Socialized by AM-Day 2 points and/or AM-Day 3 points. However, only 12 cats were misclassified by both (only 3 by more than 3 points), with 17 misclassified by AM-Day 2 (only 3 by 3 or more points) and 16 misclassified by AM-Day 3 (only 5 by 3 or more points). Correlations between Socialization Scores, points, and Strong and Weak behaviors from the Behavior Checklist are shown in Table 2. Socialization Score correlated somewhat more strongly with the two sets of points than with Weak or Strong behaviors. There were 124 cats that were classified as More Socialized based on both Strong and Weak behaviors and 16 were classified as More Socialized only by Strong or only by Weak behaviors.

**Table 2:** Spearman rank sum correlations between the results of the Behavior Checklist, AM-Day 2 and AM-Day 3 model points. Results in the upper right are from the People For Animals data set. Results in the lower left are from the Humane Alliance data set. Three cats were removed from AM-Day 3 from study due to illness.

	Socialization Score (n = 297)	Strong (n = 297)	Weak (n = 297)	AM-Day 2 points (n = 297)	AM-Day 3 points (n = 291)
Socialization Score		0.565 (95% CI: 0.482 to 0.637)	0.565 (95% CI: 0.482 to 0.637)	0.734 (95% CI: 0.677 to 0.783)	0.818 (95% CI: 0.776 to 0.853)
Strong (n = 250)	0.462 (95% CI: 0.358 to 0.554)		0.868 (95% CI: 0.836 to 0.893)	0.699 (95% CI: 0.636 to 0.753)	0.771 (95% CI: 0.720 to 0.814)
Weak (n = 250)	0.527 (95% CI: 0.431 to 0.611)	0.850 (95% CI: 0.812 to 0.881)		0.734 (95% CI: 0.677 to 0.783)	0.818 (95% CI: 0.776 to 0.853)
AM-Day 2 points (n = 250)	0.354 (95% CI: 0.241 to 0.458)	0.544 (95% CI: 0.450 to 0.626)	0.609 (95% CI: 0.524 to 0.681)		0.600 (95% CI: 0.521 to 0.669)
AM-Day 3 points (n = 250)	0.387 (95% CI: 0.276 to 0.488)	0.696 (95% CI: 0.626 to 0.755)	0.544 (95% CI: 0.450 to 0.626)	0.443 (95% CI: 0.337 to 0.537)	

### 3.3. Validation of Model Results

As above, for both the AM-Day 2 and AM-Day 3 models run using the HA data set, the PM-Day 1 cats classified as More Socialized by the Behavior Checklist were excluded. For AM-Day 2, there were 207 observations included with sensitivity of 73.7% and specificity of 64.0% and AUC of 0.7691. For the AM-Day 3, there were 185 observations (alertness could not be included since all cats were alert) with sensitivity of 76.6%, specificity of 53.9% and AUC of 0.7125. 

There were 28 cats classified as More Socialized by the Behavior Check list. Using the points from the PFA models, there were 2 (1.3% 95% CI of 0.2–4.7%) More Socialized cats missed by the Behavior Checklist and both sets of points. Neither of these cats showed any additional More Socialized behaviors during PM-Day 3.

Using the AM-Day 2 model points, there were 60 out of 93 (65%) Less Socialized cats classified as More Socialized (excluding the cats classified as More Socialized by the Behavior Checklist on PM-Day 1); 17 also were considered to be socialized based on the Checklist. There were 31 out of 119 (26%) More Socialized cats misclassified as Less Socialized by AM-Day 2 points; however, all but 3 of these cats had 12 or more points on AM-Day 3 and 19 were considered to be socialized by the Checklist. Using the AM-Day 3 model, there were 56 of 91 (62%) Less Socialized cats classified as More Socialized (excluding the cats classified as More Socialized by the Behavior Checklist on PM-Day 1 and AM-Day 2 points > 31) and 15 out of 94 (16%) More Socialized cats misclassified as Less Socialized; however all but 2 of these cats had 20 or more points on AM-Day 2 and 8 were considered to be socialized based on the Behavior Checklist. Correlations between the Behavior Checklist, AM-Day 2 points and AM-Day 3 points were somewhat lower than for the PFA data set (Table 2).

## 4. Discussion

The objective of this project was to develop a tool that could separate More Socialized but frightened cats from cats that are Less Socialized to humans within an animal shelter setting. We wanted the tool to be simple enough to be executed effectively by individuals with minimal training and those with little expertise in cat behavior. The tool also needed to be practical in an individual cage setting, safe for the cats and the humans, and be useful within a three day period after the cat arrived at the shelter. Therefore, there were constraints around the types of measures we could use to create this tool. 

Some information on reducing stress in cats in shelter settings informed our protocols and supports the likely efficacy of the tool in shelters. Having a shelf in the cage and time to relax influenced the cat’s stress and behavior in shelters and boarding catteries [3,12,13]. Furthermore, having consistent handling of individually-housed socialized cats in a reassuring way resulted in lower stress, less likelihood of euthanasia and increased likelihood of adoption in a shelter setting [13]. These findings aided our selection of required cage contents and the use of quiet, consistent interactions with all cats. In addition, we selected a three day period for observations due to a previous survey showing that about half of responding shelters were able to hold cats [5] for at least three days and that some reduction in stress would be expected during that time period [12].

We had hoped that there would be some measures that would separate the fear exhibited by More Socialized cats due to the situation (being in a shelter environment) from the fear that was exhibited by feral cats due to their fear of humans as well as the situation. We were not able to find measures to separate these two types of fear. What we did find was that More Socialized cats displayed different and more behaviors than the Less Socialized cats over time and with interaction. In addition, some More Socialized cats had certain measures that could be used to identify them. Therefore, the tool works by process of elimination during the three day period: More Socialized cats show some key measures (both obvious and subtle) while Less Socialized cats do not. For example, in addition to affiliative measures such as rubbing or kneading, More Socialized cats were more likely to be at the front of the cage more often and had their tail up at the end of an assessment. 

While we found no unique responses that only Less Socialized cats show, when statistically comparing Less Socialized and More Socialized cats, Less Socialized cats tended to be more focused on what was being done by the observer. This led to more instances of alertness, less blinking and more eye contact. 

Affiliative behaviors (or those that have been considered to be affiliative) were not exclusively expressed by More Socialized cats in our study. Some behaviors considered to be affiliative were seen exclusively in More Socialized cats while others were predominantly exhibited by More Socialized cats, but were also exhibited by Less Socialized cats. Sniffing is considered to be an affiliative behavior, something that cats do in greetings and social interactions [14]. However, we found that quite a few Less Socialized cats did sniff, especially when the rod was presented. Even more importantly, some More Socialized cats showed no Strong or Weak attention seeking behaviors even though these included affiliative behaviors. It seems likely that fear and general arousal may mask these behaviors in some More Socialized cats. Therefore, reliance only on affiliative behavior as an indicator of socialization with people would lead to some false positives as well as many false negatives. 

The use of regression modeling to develop equations to predict outcomes or to generate points is not new [15,16,17,18]. Using a logistic regression model to develop a point system for prediction provides a relationship not only between Socialization Score and the different measures in the model but also among the different measures. The points, therefore, can be used to help rank a cat’s socialization using the measures that are the best predictors. The measures which were most strongly predictive of socialization during the AM-Day 2 assessments were focusing attention on the toy, licking lips/nose, sniffing the rod during the first presentation, two measures of affiliative behavior and eating during the night. Head facing forward for the Interactive Toy was also significantly associated with being More Socialized 

The AM-Day 3 model excluded not only cats considered to be socialized based on the Checklist during PM-Day 1 but also cats with high points on AM-Day 2. Our reasoning was to remove from further logistic regression analyses the cats that were showing the most obvious indicators of socialization. This allowed the AM-Day 3 model to focus on differentiating the harder to separate, shyer and more reserved cats. In the AM-Day 3 model, More Socialized cats behaved differently as demonstrated by being less likely to have prolonged eye contact, alertness, or be in the back of the cage. The model also included affiliative behaviors during two assessments and sniffing the rod as significant predictors. The one measure that seemed counter intuitive at first was withdrawal at any time during the Crack Cage Door assessment. Here, More Socialized cats were more likely to withdraw, but not be at the back of the cage. This was determined to be due to their approaching and then withdrawing (and therefore moving), sometimes repeatedly, during the assessment.

The higher correlations between Socialization Score and the sets of Points indicates that the points are reflecting a range of socialization, and that more points does tend to equate to a higher level of socialization. This could be partly due to using the model which includes Socialization Score as the outcome variable in calculating the Points. The lower correlations for Socialization Score and Strong or Weak behaviors from the Behavior Checklist might be due to Strong or Weak behaviors reflecting only one or two aspects of what the Socialization Score measures. Strong and Weak behaviors were strongly correlated with each other indicating that they could be measuring the same component of the Socialization Score.

The higher frequency of the HA data set predicting that Less Socialized cats were socialized (using either the point systems or the Behavior Checklist) could be at least partly due to caregivers scoring somewhat socialized cats as feral to claim the $10 incentive offered for more feral cats. In addition, it is possible that the caregivers from that region viewed their cats as less socialized than caregivers from the region where the PFA cats came from. Despite these differences, we believe that the predictive models showed adequate validity and AUC. It is not possible to characterize the shelter cat population in the studied regions, or in the U.S. in general. However, we believe that our varied recruiting strategies, the cat participation incentives, the information we gained from the survey and conversations with caregivers at intake, the range of methods caregivers used to transport the cats for the study and the range of feline behaviors observed during the study all support the likelihood that the study population did reflect the typical shelter cat population.

## 5. Conclusion

We were able to predict Less Socialized and More Socialized cats using points developed from logistic regression models during two morning time periods. We found that assessments done on the first day a cat arrived at a shelter-like environment only provided information about that cat’s socialization level if the cat was very confident and demonstrative. The assessments and measures studied here are safe for the cats and humans, simple enough for most people to complete effectively, and can be done in a shelter environment. We have found that over a three day period, More Socialized cats show certain key measures that Less Socialized cats do not, and through a process of elimination, these measures can separate out More Socialized cats. There are also some behaviors that are unique to More Socialized cats even though not all More Socialized cats demonstrate these behaviors. We are planning additional testing in animal shelters to develop the final version of the tool. 
